# Cardiovascular diseases monitoring: lessons from population-based registries to address future opportunities and challenges in Europe

**DOI:** 10.1186/s13690-018-0283-3

**Published:** 2018-06-28

**Authors:** Luigi Palmieri, Giovanni Veronesi, Giovanni Corrao, Giuseppe Traversa, Marco M. Ferrario, Giovanni Nicoletti, Anna Di Lonardo, Chiara Donfrancesco, Flavia Carle, Simona Giampaoli

**Affiliations:** 1 Department of Cardiovascular, Dysmetabolic and Aging-associated Diseases, Istituto Superiore di Sanità (ISS), Via Giano della Bella, 34 00162 Rome, Italy; 2 Research Centre in Epidemiology and Preventive Medicine, Università dell’Insubria, Varese, Italy; 3 Department of Statistics and Quantitative Methods, Università di Milano-Bicocca, Milan, Italy; 4 National Centre for Drug Research and Evaluation, Istituto Superiore di Sanità (ISS), Rome, Italy; 5 Ministry of Health, Rome, Italy; 6 Department of Biomedical Sciences and Public Health, Università Politecnica delle Marche, Ancona, Italy

**Keywords:** Population-based registries, Cardiovascular diseases, Current administrative health databases, Record linkage, Time trends

## Abstract

**Background:**

Population-based registries implement the comprehensive collection of all disease events that occur in a well-characterized population within a certain time period and represent the preferred tools for disease monitoring at a population level. Main characteristics of a Population-based registry are to provide answers to defined research questions, also related to clinical and health policy purposes, assuring completeness of event identification, and implementing a process of case adjudication (validation) according to standardised diagnostic criteria.

**Methods:**

The application of a standard methodology results in the availability of reliable and comparable data and facilitates the transferability of health information for research and evidence-based health policies. Although registries are extremely useful, they require considerable resources to be implemented and maintained, high cost and efforts, to produce stable and reliable indicators.

**Results:**

Thanks to available health information and information technology, current administrative databases on hospital admissions and discharges, medication use, in-patient care utilization, surgical operations, drug dispensations, ticket exemption and invasive procedures are increasingly available. They represent basic sources of information for implementing Population-based registries.

Main strengths and limitations of Population-based registries are described taking into consideration the example of cardiovascular diseases, as well as future challenges and opportunities for implementing Population-based registries at European level.

**Conclusions:**

The integration of population-based registries and current administrative health databases may help to complete the picture of the disease rebuilding the evolution of the disease as a continuum from the onset to the possible consequent complications.

## Background

Recently published, the WHO Global Action Plan for the prevention and control of Non Communicable Disease-NCD 2013–2020 recommends a 25% reduction premature mortality from cardiovascular disease (CVD), cancer, diabetes, and chronic respiratory diseases strengthening national surveillance and monitoring system, including improved collection of data on risk factors, morbidity and mortality [1].

Population-based registries (PBRs), i.e. the comprehensive collection of all disease cases that occur in a well-characterized population (generally defined according to age, gender and geographic residency) within a certain time period, are among the preferred tools for disease monitoring at a population level. PBRs are characterized by the following main features: i) the implementation in a defined population of a reasonable size, in order to provide answers to specific research questions (http://www.cuore.iss.it/eng/bridge/pdf/WP8-D8.2_Population based registries_2017-10-30.pdf); ii) the process of case ascertainment, often integrating several data sources including mortality and hospital admission records, which should ensure the identification of all events independently from their clinical characteristics (completeness); and iii) the process of case adjudication (validation) according to diagnostic criteria. The main aims of a PBR are to evaluate the frequency, distribution and prognosis of the disease in the general population, by providing incidence, case fatality and survival rates; to evaluate time trends and changing pattern of the disease; and to monitor prevention programs. In the 1990s, the WHO-MONICA (WHO Multinational MONItoring of trends and determinants in CArdiovascular disease) project provided long-term CVD trends in different European populations using a common registry protocol which yielded to comparable estimates across populations and time periods [2]. MONICA findings suggested that the observed decline in Coronary Heart Disease (CHD) mortality was mainly attributable to a change in coronary event rates, rather than to changes in survival. More recent trends (up to 2010) in CHD attack rates, in-hospital and out-of-hospital case-fatality by sex and age group in six European populations suggested that pre-hospital CHD case-fatality declined only in selected populations and differently by gender [3]. Therefore, in the past, PBRs provided information of paramount importance for the scientific community, as well as for the clinical setting and for health policy purposes [4, 5]. However, at present reliable and comparable estimates of CVD incidence and case-fatality in the adult population, regularly elaborated and produced according to a common standard protocol, are lacking at a European level. Annual epidemiological CVD updates do not integrate mortality and hospital admission records, and therefore do not provide estimates of disease incidence, attack rates, and case-fatality [6, 7]. They are of limited use, not only for health care and health services planning, but also in primary prevention clinical practice for the updating and re-calibration of CVD risk scores (e.g. SCORE Risk Charts http://www.escardio.org/Guidelines-&-Education/Practice-tools/CVD-prevention-toolbox/SCORE-Risk-Charts). In addition, disease estimates derived from healthcare databases may suffer from lack of consistency in diagnosis coding across Countries and time periods. Similarly, CVD mortality trends are regularly released in Europe; however, CVD morbidity trends are mostly limited to the results from independent registries [7].

In this paper we describe main characteristics of population-based registers for cardiovascular diseases, strengths and limitations, by using examples from their historical evolution. In addition, we discuss future opportunities and challenges related to the implementation of PBRs at a European level.

## Methods

### Definition and main activities of a PBR

The term ‘registration’ implies the identification of all disease cases occurring in a population, during a certain time period. The main activities of a PBR involve case ascertainment, often using consolidated administrative archives as sources of event notification; the recording of clinical data for identification purposes; case adjudication according to available clinical information; case follow-up, with the collection of subsequent data on the course of the disease and relapses; and the statistical analysis of the obtained data [8]. All these activities should be planned to answer to specific research questions and objectives, and described in a registry protocol or manual [4].

### PBRs: Purpose and rationale

The first step in planning a registry is the formulation of a clearly defined purpose and rationale; this makes easier to evaluate whether the registry is the right approach to obtain the data of interest [9, 10]. A defined purpose helps to clarify the data needed. Attempts to produce an all-inclusive registry may add cost but not value, resulting in an overly data collection that reduces quality and completeness. The value of a registry must be examined at intervals to ensure that the objectives are still relevant and are met. If the registry has several purposes, these should be translated into specific objectives [11]. This process needs to take into account the interests of researcher, stakeholders, and policy makers. Clear objectives are essential to define data collection and to ensure that the registry addresses the important issues (http://www.cuore.iss.it/eng/bridge/pdf/WP8-D8.2_Population based registries_2017-10-30.pdf).

The first experience of a population-based registry in the field of CVD was the WHO Myocardial Infarction Community Registers in 1967 [12]; it was implemented by a group of experts convened by the WHO Regional office for Europe to (a) evaluate the extent of acute myocardial infarction (AMI) in the community; (b) monitor the effect of changes in the management of AMI and different types of intervention; (c) provide an assessment of the validity of mortality statistics; (d) select a pool of patients who could be studied in detail and focus attention on specific problem areas. In order to obtain a statistically sufficient number of notifications, it was decided that the duration of the registration should cover one year with a 12 months follow-up after the heart attack; each community had to be well defined demographically as census data were indispensable for establishing incidence. All persons in whom there was “any suspicion” that AMI (on the basis of history, electrocardiogram (ECG), enzyme and post mortem results) might have occurred in population who were ≤ 65 years old at the onset of the acute attack and who were resident in the registration area were admitted to the registry. The cut-off point of 65 years was chosen in order to keep the registry to a size that was easy to handle and to exclude older patients with multiple pathologies. The registry examined the incidence of AMI and the influence of smoking, obesity and hypertension on AMI to show which people in the community were specifically at risk.

The WHO Myocardial Infarction Community Registers were followed by the WHO MONICA Project (MONItoring Trends and Determinants in CArdiovascular Disease) [13] which was designed to answer key questions arising from the 1978 Bethesda Conference on the Decline in Coronary Heart Disease Mortality: ‘*are reported declines in coronary heart disease mortality genuine? If they are, how much is attributable to improved survival rather than to declining coronary-event rates? Are these trends related to changes in risk factors and health care?*’ [13]. It was a very wide project conducted between the half of ‘80s and the half of ‘90s overall the world that allowed, for the first time, (a) to collect and register, during 10-year surveillance of 37 populations in 21 countries, 166,000 first and recurrent events in men and women aged 35-64 years; (b) to classify, following the same standardised diagnostic criteria (site and duration of pain, evolution of ECG findings, variation of cardiac enzyme values and history of Ischaemic Heart Disease (IHD), and, if performed, necropsy), all suspected events in fatal and non-fatal ‘definite’, ‘possible’, ‘ischemic cardiac arrest with successful resuscitation’, and ‘insufficient data’ events. An important improvement in the use of standardised diagnostic criteria was the introduction of a quantitative ECG coding system, the Minnesota Code [14]. Main results of the WHO MONICA Registry demonstrated that contributions to changing of IHD mortality varied, but in populations in which mortality decreased, coronary event rates contributed for two thirds and case fatality for one-third [2]. These trends were related to changes in known risk factors (systolic blood pressure, total cholesterol, smoking habit and body mass index) daily living habits, health care and major socio-economic features measured at the same time in defined communities in different countries [13].

The European Cardiovascular Indicators Surveillance Set (EUROCISS) Project was launched in 2000 by a partnership of 14 EU countries; many of the partners were collaborating centres of the MONICA Registry and had actively continued the registration of cardiovascular events. The aim of the project was, starting from the MONICA experience, to develop health indicators and recommendations for monitoring the distribution and impact of CVD in Europe in order to facilitate cross-country comparisons and improve CVD prevention. An updated picture of the existing population-based registries of AMI and stroke in Europe was published, with a detailed description of sources of information, data collection, validation methods and indicators assessed in the different population-based registries [15]. Even though deriving from the MONICA experience, these registries basically were not comparable since they collected and validated events with different characteristics (population size, age-range under surveillance, International Classification of Diseases-ICD codes used to identify suspected events, validation procedures) [15]. AMI incidence/attack rate, case fatality and prevalence were suggested for inclusion in the European Community Health Indicators (ECHI) short list (n.24 and 25) [16]. A second phase of the EUROCISS Project (2004–2007) was launched aiming at (a) developing knowledge, tools and expertise among Member States for CVD surveillance and prevention; (b) preparing the Manual of Operations for the implementation of a population-based registry of AMI/acute coronary syndrome (AMI/ACS) [17], of stroke [18], and of CVD surveys for assessing standardized indicators (prevalence of old myocardial infarction, heart failure, late effects of cerebrovascular diseases, and other CVD), and for identifying a minimum set of questions and exams to be included in the Health Examination Survey (HES) for evaluating the prevalence of CVD at European level [19]. The EUROCISS project provided recommendations to assess reliable and comparable indicators, methodologies for validating suspected cases and for classifying events, and suggested a stepwise procedure to assess the indicators proposed and recommended for the ECHI list.

At national level, a pilot study for the population-based Italian Registry of CVD was implemented covering fatal and non-fatal coronary and cerebrovascular events in the general population aged 35–74 years. It was launched in Italy on 2000 following the MONICA and EUROCISS experiences with the aim of estimating periodically attack rates and case fatality rates of coronary and cerebrovascular events in geographical macro-areas representative of the country, in order to monitor time trends of CVD of major impact in adult population and assess the geographical gradient North-South [20, 21]. Some questions to which the pilot study for the population-based Italian Registry of coronary an cerebrovascular events intended to answer included, for example: – what is the frequency of AMI and who are the persons at particular risk?; − are there any difference in the incidence of AMI between North and South of Italy?; − what proportion of people suffering from AMI recovers satisfactorily and what proportion dies?; − where does that occur, at home or in a hospital?; − what happens to patient after leaving hospital?

## Results

### Case ascertainment and evaluation of registry completeness

The process of case ascertainment aims at the identification of all disease cases, independently from their clinical characteristics.

#### Sources of event notification

In the WHO MONICA project, case findings were obtained by either *hot pursuit* or *cold pursuit*. *Hot pursuit* means identifying case admissions to hospital usually within one or two days of event onset and acquiring relevant information by visiting the ward or interviewing the patient. *Cold pursuit* implies the identification of cases from final diagnosis (i.e. hospital discharge diagnosis), and the collection of clinical data by case not abstraction and review of available clinical information. The *cold pursuit* process is easier and less expensive than hot pursuit; the number of cases studied is typically smaller because discharge diagnoses are more precise and specific than those on admission. Nowadays, the main sources for case ascertainment are current administrative health databases, also denoted as HealthCare Utilization (HCU) databases, which include current data on hospital admissions and discharges, in-patient care utilization, drug prescriptions, outpatient visits, exemption, general practitioner (GP) databases, surgical operations and invasive procedures.

#### Integration of multiple sources of event notification

**Fig. 1:**
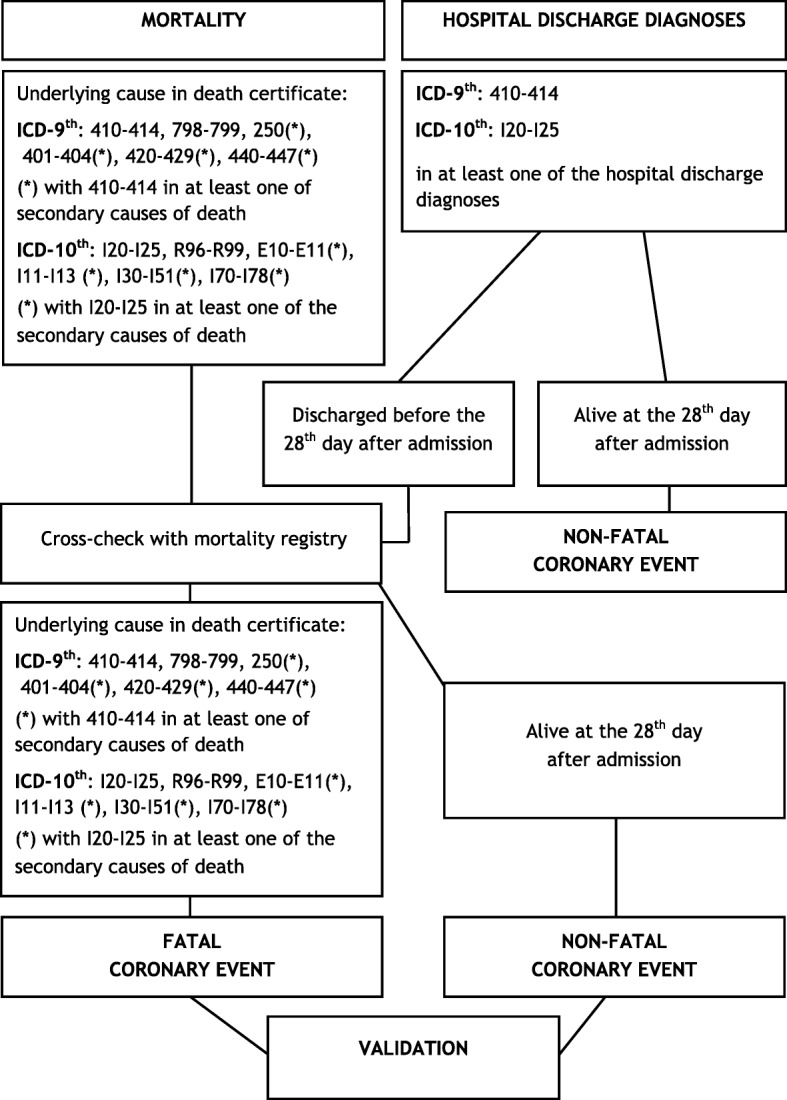
Flow-chart to select fatal and non-fatal *coronary* events in the Italian Registry of Cardiovascular Diseases

The use of only one data source is often not enough for the purposes of a complete data ascertainment. For instance, in an acute condition such as myocardial infarction or stroke, pre-hospital deaths still represent a significant proportion of overall fatal cases (around 30%). These would be lost (not registered) if the hospital admission database were they only source of data [2, 22]. With exemplification purposes, we report in Fig. 1 the diagnosis codes adopted in the pilot study for the population-based Italian Registry of CVD to select suspected fatal and non-fatal coronary events from the mortality and hospital discharge records databases. In chronic conditions, such as asthma, chronic obstructive pulmonary disease, obstructive lung diseases and heart failure (HF), a surveillance program in Italy integrates up to 5 different electronic sources (death certificates, hospital discharge records -including outpatient discharges-, drug prescriptions, tax-exemptions, and pathology records) [23]. Integration of different data sources is made possible through record linkage using a unique subject identification code. Advanced record linkage methods are also available to account for possible errors/uncomplete registrations of the identification code in HCUs [24]. Finally, the integration of data sources through record linkage is also necessary to avoid double counting of events, such as deaths in hospital for which both hospital record and death certificate are available; or successive hospital admissions in a short time period due to the same acute event onset.

#### Coverage

To ensure data completeness, it is important that all cases concerning the residents of a defined area are recorded, even when the case occurs outside the person’s area of residence. In the same way, all cases treated at hospitals located in a specific area but involving patients who reside outside that area must be excluded.

#### Assessment of completeness in data ascertainment

Completeness in case ascertainment can be measured as part of PBRs data quality assessment [4], by comparing the number of cases included into the registry with the number of cases from HCUs. For instance, in a coronary heart disease register, the ratio between the number of fatal cases in the register and the number of coronary deaths from routine mortality data should be larger than 1, to indicate that register has investigated more fatal cases than those present in mortality database [25].

### Case adjudication (validation) according to diagnostic criteria

The use of standard epidemiological criteria to define a case is called case adjudication or validation. Event validation is considered an important strength of population-based registries [26, 27]. In fact, validation provides the means to take into account bias from diagnostic practices and changes in coding systems; it traces the impact of new diagnostic tools and re-definition of events; it ensures data comparability within the registry (i.e. different sub-populations, different time points, etc.) as well as with other registries within and between Countries. For instance, in MONICA registries, coronary events were adjudicated applying the MONICA diagnostic criteria, which classified the event as ‘definite’, ‘possible’, ‘ischemic cardiac arrest with successful resuscitation’, and ‘insufficient data’ based on presence and duration of symptoms, ECG read by Minnesota code, cardiac enzymes, history of IHD, and autopsy data (http://www.thl.fi/publications/monica/manual/part4/iv-1.htm). In addition, event duration was fixed at 28 days, i.e. if the same patient experienced further a second acute onset within 28 days after a first one, the second episode was not counted as a new event [13].

Here following, we provide three examples to illustrate the importance of case adjudication. In a PBR of acute coronary events in the Varese Province (Northern Italy), about 70% of death certificates reporting a ICD code 414 (other forms of chronic ischemic heart disease) as underlying cause of death were adjudicated as coronary heart disease events according to the MONICA definition [25]. Had all these deaths been included as events due to lack of validation, the registry would have been included a number of false positives. Since this proportion may change over time and across populations, this may affect temporal trends estimates and geographic comparisons [25].

The second example illustrates how validation is crucial especially when diagnostic criteria change during time and new and more sensitive instrumental examinations are increasingly implemented. In a study on ischemic strokes based on data collected from a Northern Italy Population-Based Register, the proportion of Ischaemic Strokes (IS) cases with reported minor deficits at onset (e.g. paraesthesia), increased between 1998 and 2004 (from 6.0 to 18.1%), while the proportion of IS cases with a reported disturbed level of consciousness, as well as the proportion of patients with paresis or aphasia among those who were conscious, decreased. In the same period, comparing the use of diagnostic imaging devices, a significant increase in the use of Magnetic Resonance Imaging-MRI (both with and without angiography) among ischemic cases (*p* < 0.001) and a parallel decreasing prevalence of Computerised Tomography-CT scan examination (employed in 99 and 91% of the total number of cases in 1998 and 2004, respectively), were registered [28]. In not appropriately accounted for by using a consistent event definition over time, these differences are likely to alter the estimates of disease trends based on discharge diagnosis alone.

Finally, as third example, in the context of health failure research, hospital discharge diagnosis (ICD-9428, ICD-10 I50) needs validation to understand whether they can be used to distinguish those who actually have HF from those who are hospitalised for different chronic morbidities. In fact, HF patients tend to have high comorbidity burdens and be hospitalized for other cardiovascular and respiratory conditions [29, 30]. While HF may have contributed to the need for these hospitalizations, this diagnosis may not be entered on the discharge record; therefore HF specific ICD codes should be detected not only in the first discharge diagnosis, but also in the secondary ones. According to a recent summary of available evidence [31], however, HF codes that do appear in administrative databases are highly predictive of true HF cases. At the same time, nevertheless, administrative databases fail to capture a non-negligible number of true cases, perhaps 25 to 30% of all diagnoses, and may differentially capture only the most severe cases.

### Strengths and limitations of population-based registries for CVD monitoring

The completeness of event identification, also due to the possibility to integrate multiple data sources, and the consistent adjudication of cases over time are the major strengths of PBRs for monitoring of cardiovascular disease at a population level. Other sources of information, such as clinical hospital-based registries, fail to provide a complete event registration, due to lack of information on pre-hospital fatal cases [2, 3, 22] and patients’ selection bias [32]. Similarly, the value of monitoring systems based upon secondary use of HCUs without event validation rests heavily on the accuracy of data for ascertaining disease cases. To reduce misclassification errors in case ascertainment, researchers often make use of case definitions (usually in the form of algorithms based on diagnosis codes and/or other information such as pharmacy dispensations). However, estimates of disease prevalence using different algorithms may vary by as much as 50% [33, 34]. As described in the HF registry example, administrative databases fail to capture between the 25% and the 30% of true cases and may differentially capture only the most severe cases.

The major limitation is that implementing a population-based registry is highly demanding in terms of time consuming, personnel and financial support. Population-based registries are expensive especially for access to data files, identification of suspected events, gathering all clinical records required to apply diagnostic criteria, implementing event validation process, which should be homogeneous across countries in order to produce representative, accurate and comparable data. The procedures of case ascertainment and adjudication require trained personnel, to collect information from clinical records and to apply standard epidemiological event definition (to involve an epidemiological team would be desirable since clinician often prefer to use subjective and qualitative judgements for event validation). Other time consuming activities are implementing and maintaining quality control standards; in case of multi-populations register, site visits are desirable to verify accurate and reliable implementation of procedures. As a consequence, PBRs may lack of timeliness in the availability of results to the scientific community, with a delay of 3 to 5 years from data collection to results publication.

**Fig. 2:**
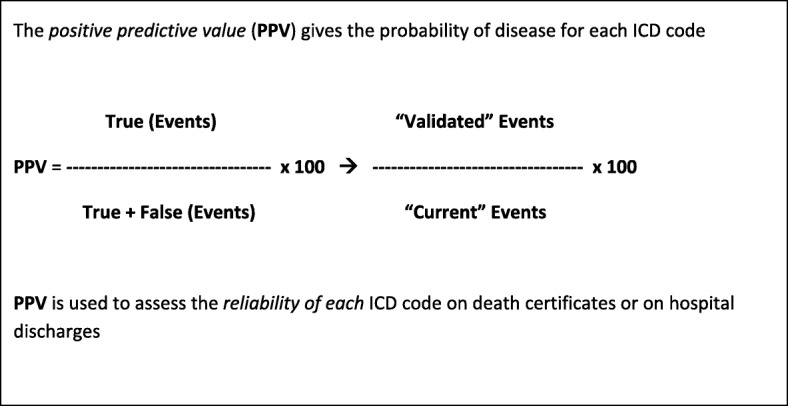
Positive predictive value for an identified ICD code in the Italian Registry of Cardiovascular Diseases

**Fig. 3:**
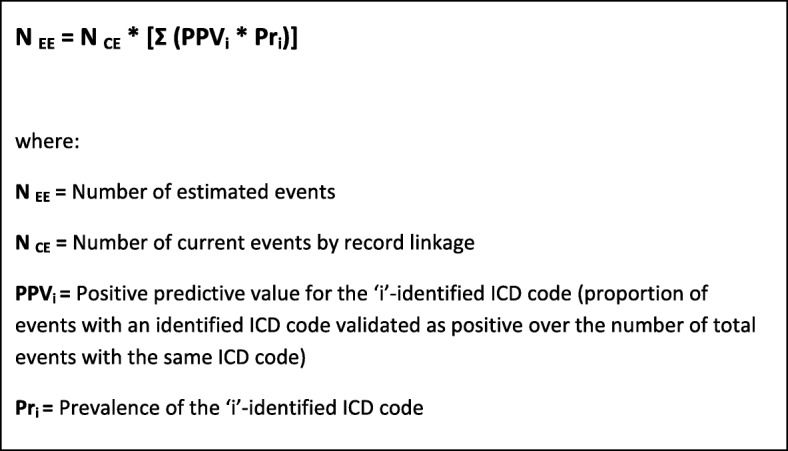
Number of estimated events for an identified ICD code (fatal and non-fatal events separately)

**Fig. 4:**
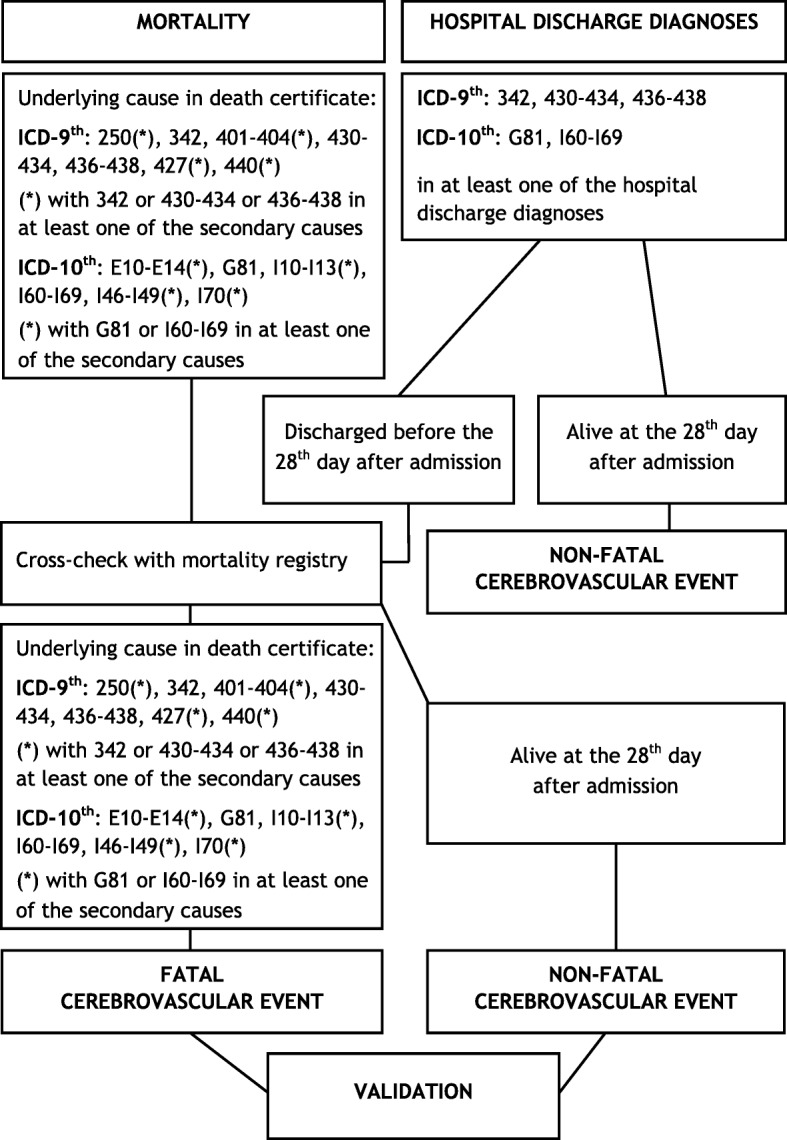
Flow-chart to select fatal and non-fatal *cerebrovascular* events in the Italian Registry of cardiovascular diseases

In order to reduce resources absorption, some measures can be taken, such as the validation of a random sub-sample of cases. In the pilot study of the national Italian Registry of coronary and cerebrovascular events, a random sample of 4000 current suspected events per year (500 events from each of the 8 registries) was validated. Figure 2 reports the Positive Predictive Value (PPV) for each ICD code of the main cause of death of fatal events, and for each ICD code of the first hospital discharge diagnosis of non-fatal events. For example, in the registry of cerebrovascular events [21], overall PPVs of ICD codes identifying suspected fatal events were 69% in men and 73% in women; this means that out of all the fatal events identified by selected ICD codes 31% in men and 27% in women were not confirmed as cerebrovascular events after validation. To calculate the number of estimated events, the number of suspected events was multiplied by the PPV of each specific mortality or discharge ICD code derived from the validation of the random sample of suspected events (Fig. 3). Attack rates, which include first and recurrent events, in the age range 35–74 years, were then calculated dividing the number of estimated events by the resident population by 10-years age groups, and standardized by direct method using the European Standard Population; case fatality rate at the 28th day was determined by the ratio between estimated fatal events and total events [20, 35]. Similar methodological path was applied for identifying fatal and non-fatal stroke events and for estimating related attack rates and case fatality in the population (Fig. 4) [21].

## Discussion

### Future opportunities and challenges related to the implementation of PBRs at national and European level

*Standardization* – As the EUROCISS project has widely demonstrated for cardiovascular disease registries, standardised procedures for the identification of events and agreed standardised diagnostic criteria for their validation are needed, even though they are not commonly adopted yet at European level [15]. If PBRs related to the same disease and implemented in different areas of the country adopt agreed and standardised procedures for data collection and standardised diagnostic criteria for event validation, they can be organised and integrated in a national research platform. This is the example of the cancer registries, unified in Italy under the AIRTUM organization (http://www.registri-tumori.it/cms/), and participating to the EUROCARE Programmes (http://cordis.europa.eu/project/rcn/46511_en.html, http://www.eurocare.it/Eurocare6/tabid/92/Default.aspx) at European level. This process will facilitate sustainability of PBRs and their continuative implementation attracting regular funds both at national (from Ministry of Health) and European level.

#### PBR integration with epidemiological studies and HCUs (interoperability)

Population-based mortality registries and health interview surveys, often managed by the National Institutes of Statistics, provide information on mortality and perceived health status; morbidity registries, epidemiological research studies, such as longitudinal studies and Health Examination Survey (HESs), usually conducted by National Institutes of Public Health, provide health information on occurrence and prevalence of disease, on measured risk factors, lifestyles, and high risk conditions by standardized methods and diagnostic criteria.

In recent years, thanks to information technology, other sources of information of health current data, managed by Health Authorities (HCU databases), are routinely collected for evaluating hospitalised complications, for administrative purposes related to reimbursement of provided services, for management of health services and healthcare expenditure, and for guaranteeing equity of health care system.

To integrate these huge sources of information allows to follow-up patients over time and to perform observational studies aimed at investigating the relationship between risk factors, healthcare pathways, their possible interactions, and selected outcomes for generating evidence on quality of care and efficacy of therapeutic and assistance pathways in relation to diagnoses.

With the aim of ensuring data interconnectability (i.e., the feasibility to recognize health services supplied to a given beneficiary of health system jointly to his/her health-related characteristics through an unique personal identifier) and platforms interoperability (i.e., the capability of heterogeneous platforms of interchanging data in a way that the data from one can be recognized, interpreted, used and processed by the others [36]), while complying legal and privacy rules, several models may be used.

A possible way is of implementing country specific research platforms able to: (i) integrate in a safe environment, data from PBRs, epidemiological studies and HCUs whose accessibility is regulated by harmonized rules in different countries; (ii) allow of extracting, storing and standardizing data deriving from different and heterogeneous sources; (iii) agree data organization by means of a protocol driven approach (i.e., extracting relevant fields, selecting records according to predefined inclusion or exclusion criteria, allowing the implementation of observational designs and statistical tools); and (iv) compare and summarize population health (incidence and survival) with healthcare indicators and evidence generated by each country according to a common protocol. Taking lessons from results of the BRIDGE project (http://www.cuore.iss.it/eng/bridge/pdf/WP8-D8.2_Population based registries_2017-10-30.pdf), European countries are addressing along this path to verify its feasibility and potentiality including the interoperability issue in the Joint Action on Health Information-InfAct, which is about to start, funded by the European Commission (https://ec.europa.eu/health/sites/health/files/indicators/docs/ev_20171206_conclusions_en.pdf, http://ec.europa.eu/chafea/health/actions.html).

*Ethical aspects* - Population-based registries for epidemiological studies contain information about the health status of a person; this is considered sensitive data by law and therefore subject to protection. The identification of each subject in the different situations (registries or use of administrative data) has generated legal and ethical problems, given that the personal integrity (autonomy, confidentiality and privacy) of individuals has to be guaranteed. In particular, consideration shall be paid to the fundamental principles applied through norms, and the latest EU Regulation (2016/679) on the protection of natural persons [37].

In the case of population-based registries, the principle of ‘respect for persons’ is well represented and supported by the “informed consent” of the subjects to the use of their health information (HI) for research purposes. A general ethical requirement for consent clearly implies that human subjects voluntarily permit the use of their HI in a registry, unless a specific exception to voluntary participation applies to the registry itself. One such exception is a legally mandated, public health justification for the compilation of HI. Voluntary agreement to the use of HI in a registry necessarily allows a subsequent decision to discontinue participation.

The consent given for a registry concerns, mainly, two different aspects:

- consent to create the registry by compilation of patient information;

- consent to the use of data for the purpose of the registry and for other declared purposes.

The issue related to ethical aspects, in particular the different way European countries incorporate and implement the recent EU Regulation, still represents a barrier to the implementation of PBRs at national level.

## Conclusion

In the field of cardiovascular diseases, PBRs provide important and reliable indicators for etiologic research, time trend, geographical gradient, and survival studies aiming at improving health care (e.g. incidence rate, case fatality, prevalence, survival), but need long time working to reach reliable and stable results and wide resident population to be maintained under surveillance.

Validation constitutes the added value of PBRs providing reliable and comparable incidence rates and other indicators in comparison to a continuous and systematic collection of available data from health sources of information (e.g. HCU databases).

Several operative limitations and challenges weaken sustainability of PBRs at national level and even more at European level; but the increasing availability of other sources of information of current health data, can represent an opportunity to facilitate the implementation of PBRs and extend the population under surveillance.

The integration of epidemiological studies, including population-based registries, and current administrative health databases can also help to fill in the lack of reliable information on the occurrence of the disease especially related to quality of healthcare and to efficacy of therapeutic and assistance pathways in relation to diagnosis.

## Abbreviations

ACS: Acute Coronary Syndrome
AMI: Acute Myocardial Infarction
CHD: Coronary Heart Disease
CT: Computerised Tomography
CVD: Cardiovascular diseases
ECG: Electrocardiogram
GP: General Practitioner
HCU: HealthCare Utilization
HES: Health Examination Survey
HF: Heart Failure
HI: Health Information
ICD: International Classification of Diseases
IHD: Ischaemic Heart Disease
IS: Ischaemic Strokes
MRI: Magnetic Resonance Imaging
PBRs: Population-based registries
PPV: Positive Predictive Value
WHO: World health Organization
